# Real-Time Extraction of Important Surgical Phases in Cataract Surgery Videos

**DOI:** 10.1038/s41598-019-53091-8

**Published:** 2019-11-12

**Authors:** Shoji Morita, Hitoshi Tabuchi, Hiroki Masumoto, Tomofusa Yamauchi, Naotake Kamiura

**Affiliations:** 1 Graduate School of Engineering, University of Hyogo, Kobe, Japan; 2 Department of Ophthalmology, Tsukazaki Hospital, Himeji, Japan

**Keywords:** Medical research, Information technology

## Abstract

The present study aimed to conduct a real-time automatic analysis of two important surgical phases, which are continuous curvilinear capsulorrhexis (CCC), nuclear extraction, and three other surgical phases of cataract surgery using artificial intelligence technology. A total of 303 cases of cataract surgery registered in the clinical database of the Ophthalmology Department of Tsukazaki Hospital were used as a dataset. Surgical videos were downsampled to a resolution of 299 × 168 at 1 FPS to image each frame. Next, based on the start and end times of each surgical phase recorded by an ophthalmologist, the obtained images were labeled correctly. Using the data, a neural network model, known as InceptionV3, was developed to identify the given surgical phase for each image. Then, the obtained images were processed in chronological order using the neural network model, where the moving average of the output result of five consecutive images was derived. The class with the maximum output value was defined as the surgical phase. For each surgical phase, the time at which a phase was first identified was defined as the start time, and the time at which a phase was last identified was defined as the end time. The performance was evaluated by finding the mean absolute error between the start and end times of each important phase recorded by the ophthalmologist as well as the start and end times determined by the model. The correct response rate of the cataract surgical phase classification was 90.7% for CCC, 94.5% for nuclear extraction, and 97.9% for other phases, with a mean correct response rate of 96.5%. The errors between each phase’s start and end times recorded by the ophthalmologist and those determined by the neural network model were as follows: CCC’s start and end times, 3.34 seconds and 4.43 seconds, respectively and nuclear extraction’s start and end times, 7.21 seconds and 6.04 seconds, respectively, with a mean of 5.25 seconds. The neural network model used in this study was able to perform the classification of the surgical phase by only referring to the last 5 seconds of video images. Therefore, our method has performed like a real-time classification.

## Introduction

Surgeons’ experience has been scientifically proven to influence postoperative results. For example, an increased risk of postoperative complications is reported for surgeons who have performed less than 500 gastric bypass surgeries compared to surgeons who have performed more than 500 surgeries^1^. It has been reported that the risk of patients developing reactive corneal edema as determined by the central corneal thickness 2 hours after surgery was about 1.6 times higher for novice surgeons performing cataract surgery than surgeons with experience^2^. Thus, shortening the learning curve of a surgical technique is reported to be one of the most important challenges in medicine.

The difficulty of setting objective indicators to evaluate surgical technique is often regarded as a problem in surgical training. It has been pointed out that measuring quantitatively and standardizing surgical techniques are required to systematically advance surgical training^3^. However, continuous curvilinear capsulorrhexis (CCC), a critical phase of cataract surgery, for example, involves several techniques^4^, and it is impossible to index and measure all of these techniques in terms of medical economics; therefore, it is not a realistic proposition.

Deep learning is a breakthrough in machine learning, and it has been applied to a number of research areas in all industries. In the field of ophthalmology as well a great number of studies on image recognition have been conducted after Google published a paper on diabetic retinopathy diagnosis in 2016^5–7^. Regarding automatic diagnostic systems of diabetic retinopathy, these technologies are in line with the principle that technology promotes the efficient use of human resources and eliminates inevitable judgment errors by humans. It is logical that machine learning methods focused on deep learning have been examined even in this unexplored area of objective evaluation of surgical techniques. In recent years, a number of studies on recognition of surgical phases using surgical video recordings have been actively conducted. Recognition of a surgical phase often employs features such as image color or features of surgical instruments, as well as methods using the hidden Markov model^8^. Cataract surgery videos have been used in numerous studies, where features were extracted based on color features, SIFT features^9^, and Viola–Jones object detection framework^10^. In one study, the hidden Markov model and a time expansion and contraction method^11^ were used to detect surgical phases^12^; another study used conditional random fields^13^, the hidden Markov model, and a time expansion and contraction method^14^. These automatic detections of surgical phases are important technologies that allow clinicians to evaluate the surgical technique for a specific phase. However, in these studies, surgical phases were only identifiable from the entire surgical video, which made real-time identification impossible; moreover, even if real-time identification was possible, similar surgeries had to be searched in a database. Although a method using recurrent neural network^15^ has been proposed, surgical phase recognition was performed using a 33-second-long video. Since a number of surgical steps are involved in a short period of time in cataract surgery, the proposed method cannot be considered real-time recognition. In^16^, frames have been considered to be targets for classification, and CNNs and recurrent neural networks (RNNs) have been applied the the classification. In terms of network structure, this method seems to allow real-time processing; however, it is not mentioned how many seconds of video were used for classification with RNNs, nor examined whether it is real-time classification. Furthermore, to the best of our knowledge, there have been no reports that evaluate how accurately a neural network model divides surgical phases in addition to calculating the correct response rate per image frame.

With cataract surgical technique evaluation in mind, the present study developed a model using a neural network to perform real-time extraction of the CCC and nuclear extraction phases, which are important surgical phases in cataract surgery, from surgical video recordings. In addition, this model can divide surgical phases accurately by comparing the start and end times of each surgical phase determined by the model with the actual times.

## Datasets

In this study, video recordings of cataract surgery (phacoemulsification) performed at Saneikai Tsukazaki Hospital, a social medical care corporation, were used for the recognition of surgical phases, with a resolution of 1920 × 1080 at a frame rate of 30 FPS and a mean duration of about 534 seconds and a standard deviation (SD) of about 237 seconds. The mean (SD) durations of each phase were as follows: about 42 (44) seconds for CCC, about 133 (85) seconds for nuclear extraction, and 359 (163) seconds for other phases. Of the 303 surgical videos, 245 videos were utilized as training data, 10 as verification data, and 48 as test data.

**Figure 1:**
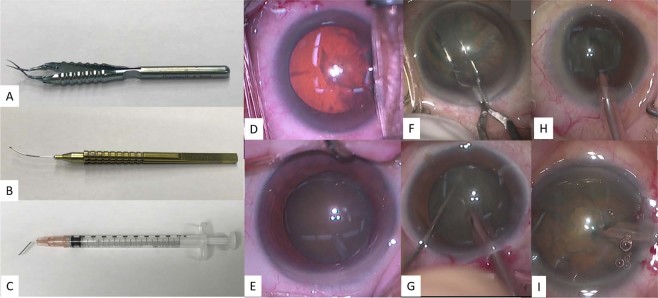
Examples of surgical instruments, lighting methods, and nuclear extraction techniques. (**A**) Inamura forceps, (**B**) Ikeda forceps, (**C**) cystotome, **(D**) retro illumination, (**E**) direct illumination, **(F**) phaco-prechopper method, (**G**) phaco-chopper method, (**H**) divide and conquer method, (**I**) central-divide method.

A total of 17 surgeons were included in 303 surgical videos. Three types of forceps were used for CCC, four types of surgical techniques were used for nuclear extraction, and two types of lighting methods were used for CCC and nuclear extraction. There were total of 9 combination patterns with these 4 variables. In Table 1, the percentage of each pattern is shown detailing the following: CCC forceps, nuclear extraction method, the lighting methods for CCC and nuclear extraction, the number of videos, and the number of surgeons. Also, photographs of these forceps, lighting methods, and surgical techniques are shown in Fig. 1.

**Table 1:** Combination patterns of surgical instruments, surgical techniques, and lighting methods.

Pattern	Name of CCC forceps	Nuclear extraction method	Lighting method (CCC)	Lighting method (nuclear extraction)	Number of videos	Number of surgeons	Percentage
No. 1	Inamura forceps	Pre-chopper	Retro illumination	Retro illumination	71	1	23.4%
No. 2	Ikeda forceps	Central-divide	Direct illumination	Direct illumination	68	1	22.4%
No. 3	Ikeda forceps	Phaco-chopper	Retro illumination	Direct illumination	51	1	16.8%
No. 4	Inamura forceps	Phaco-chopper	Retro illumination	Direct illumination	49	1	16.2%
No. 5	Inamura forceps	Phaco-chopper	Direct illumination	Direct illumination	35	8	11.6%
No. 6	Inamura forceps	Divide and conquer	Direct illumination	Direct illumination	12	2	4.0%
No. 7	Inamura forceps	Central-divide	Direct illumination	Direct illumination	7	1	2.3%
No. 8	Ikeda forceps	Divide and conquer	Direct illumination	Direct illumination	7	1	2.3%
No. 9	Cystotome	Divide and conquer	Direct illumination	Direct illumination	3	1	1.0%
**9 patterns**	**3 patterns**	**4 patterns**	**2 patterns**	**2 patterns**	**303 cases**	**17 surgeons**	**100%**

The study was approved by the Ethics Committee of Tsukazaki Hospital (Himeji, Japan) and was conducted in accordance with the tenets of the Declaration of Helsinki. Since this study only reviewed the surgical videos retrospectively and there were no anonymous issues involved, the Institutional Review Board of Tsukazaki Hospital waived the need for consent.

**Figure 2:**
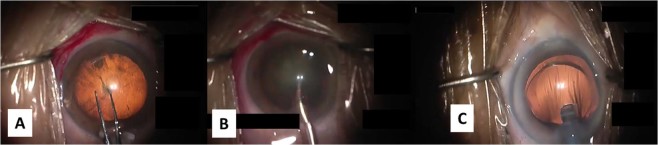
Sample images of three surgical phases. (**A**) CCC (Inamura forceps, retro illumination method), (**B**) nuclear extraction, (**C**) others (intraocular lens insertion).

Videos were downsampled to a resolution of 256 × 168 at 1 FPS in order to perform surgical phase recognition for each image. As a result, a total of 161,140 images were obtained from 303 videos. The surgical phases were correctly labeled as CCC, nuclear extraction, and others. The labels were given based on the start and end times of each surgical phase recorded by an ophthalmologist. Table 2 lists the number of image datasets obtained for each surgical phase, and Fig. 2 shows sample images of actual surgery showing three phases.

**Table 2:** Breakdown of dataset for recognition of cataract surgical phases.

Recognition class	Training data (images)	Validation data (images)	Test data (images)	Total (images)
CCC	10719	211	1725	12655
Nuclear extraction	33020	976	5995	39991
Others	90023	2376	16095	108494
Total	133762	3563	23815	161140

## Methods

### Surgical phase recognition with inception V3

**Figure 3:**
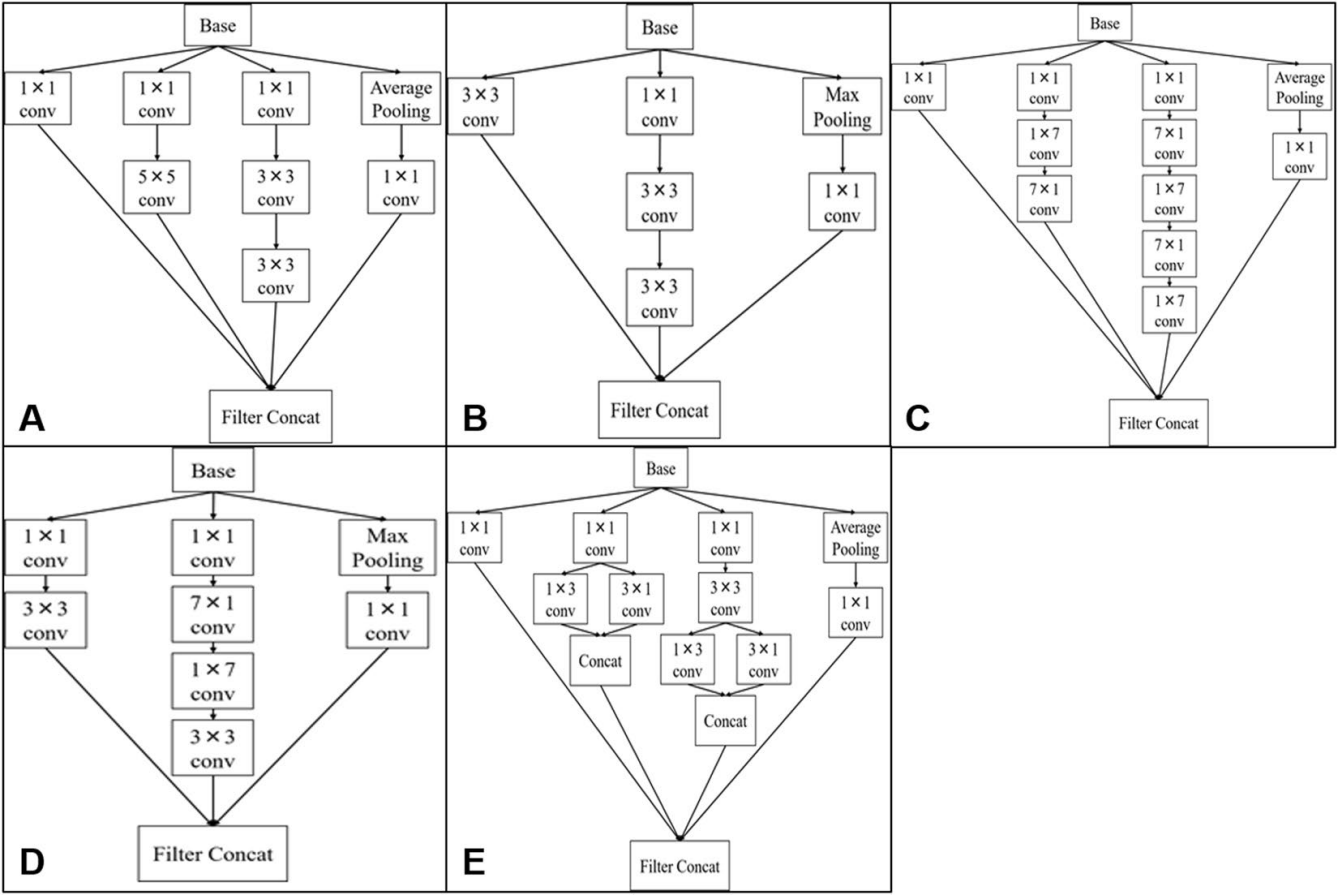
The architectures of deep neural networks. (**A**) Inception module using 5 × 5, 3 × 3, and 1 × 1 convolution. It replaces the 5 × 5 convolution layer. (**B**) Inception module using 3 × 3 and 1 × 1 convolution. It replaces the 3 × 3 convolution layer. (**C**) Inception module using 1 × 7, 7 × 1, and 1 × 1 convolution. It replaces the 7 × 7 convolution layer. (**D**) Inception module using 3 × 3, 1 × 7, 7 × 1, and 1 × 1 convolution. It replaces the 7 × 7 convolution layer. (**E**) Inception module using 3 × 3, 1 × 3, 3 × 1, and 1 × 1 convolution. It replaces 3 × 3 convolution layer.

In the present study, a convolution neural network model, known as the Inception V3 model^17^, was used to recognize three surgical phases. The Inception module was adopted in reducing the amount of computation and suppressing gradient elimination by replacing n × n convolution with 1 × n convolution and n × 1 convolution. Figure 3(A–E) illustrate the Inception module used in this study to develop the surgical phase recognition model. “Base” refers to an input tensor for an Inception module. Convolution (denoted by “conv”) makes it possible to learn high level features of an image. The convolution operation is then performed in a local region. Pooling compresses data and downsamples to reduce computational cost and suppress over-learning. Max Pooling compresses data by calculating the maximum value associated with the local region, whereas Average Pooling compresses data by calculating the average value associated with the local region. Filter concatenation (“Filter Concat”) connects multiple tensors.

As shown in Table 3, an Inception V3 model was developed to classify images into three surgical phases. Type column lists the type of layer, and patch size/stride refers to the window size and the stride of the sliding window size of a local region. The input shape column lists the size of a tensor input into each layer. The model performs computation from the top to the bottom of the table. The input was a color image of 299 × 168 × 3, and the number of output layer neurons was 3, which is the number of surgical phases to recognize. The class to which the neuron with the largest output value belonged was determined as a surgical phase of the model. The model was trained by initializing each parameter with trained parameters in the ILSVRC 2012 dataset^18^. The batch size was 32, the loss function was Multi-class log loss, the optimization function was Momentum SGD (learning rate, 0.0001; momentum, 0.9), and the number of epochs was 300 at maximum. In addition, images were preprocessed to normalize the pixel values ranging from 0 to 1, and the preprocessing steps were randomly applied in order to prevent overlearning, as shown in Table 4. In addition, the problem that classification classes are biased due to imbalanced data in each class was addressed by letting the model learn the data of the minor class within one epoch multiple times.

**Table 3:** The Inception V3 model.

Type	Patch size/stride	Input shape
Convolution	3 × 3/2	299 × 168 × 3
Convolution	3 × 3/1	149 × 83 × 32
Convolution padded	3 × 3/1	147 × 81 × 32
Max pooling	3 × 3/2	147 × 81 × 64
Convolution	1 × 1/1	73 × 40 × 64
Convolution	3 × 3/1	73 × 40 × 80
Max pooling	3 × 3/2	71 × 38 × 192
Inception	As in Fig. 3(A)	35 × 18 × 192
2 × inception	As in Fig. 3(A)	35 × 18 × 256
Inception	As in Fig. 3(B)	35 × 18 × 288
4 × inception	As in Fig. 3(C)	35 × 18 × 288
Inception	As in Fig. 3(D)	17 × 8 × 768
2 × inception	As in Fig. 3(E)	8 × 3 × 1280
Average pooling	8 × 3	8 × 3 × 2048
Full connection		2048
Full connection		1024
Softmax		3

**Table 4:** Preprocessing randomly applied to images.

Types	Parameters
Rotation	Up to 90 degrees
Horizontal movement	Up to 20%
Vertical movement	Up to 20%
Shear conversion	Up to 5 degrees
Scaling	Up to 10%
Channel shift	Up to 100
Flip horizontally	
Flip vertically	
Random erasing^20^	Up to 25%

### Extraction of important surgical phases with inception V3

Next, the start and end times of each surgical phase were determined using the Inception V3 model described in Section 3.1. Because this study put importance on real-time classification, the start and end times of surgical phases were determined using the moving average instead of a neural network. In addition, having more frames for the moving average would increase the number of images to be referenced just prior to the classification, resulting in delayed response. For this reason, the moving average was obtained using 5 frames. First, surgical images were arranged in chronological order and processed using the Inception V3 model. There are three neurons in the output layer. They correspond to three classes (i.e., CCC, nuclear extraction, and others). Let *eval*_*i*_^*j*^ denote the value of the *j*-th output neuron at the *i*-th second, where 1 ≤ *j* ≤ 3. A moving average of the values associated with each of the three output neurons is calculated from 5 consecutive images. It is denoted by *Ave*_*i*_^*j*^. It is as follows.

1 $$Av{e}_{{i}^{j}}=\frac{1}{5}\mathop{\sum }\limits_{k=i-4}^{i}\,eva{l}_{{k}^{j}}$$

where i > 4. The frame at the ith second is classified by calculating the maximum of three values, *Ave*_*i*_^1^, *Ave*_*i*_^2^, and *Ave*_*i*_^3^. In other words, the calss of the frame is specified by the neuron with the maximum of three output values. The proposed method considers the time when a frame is first judged as CCC class to be the start time of CCC, whereas considers the time immediately before a frame is first judged as nuclear-extraction class to be the end time of CCC. The start and end times of the nuclear extraction were also determined in the same manner.

## Results

### Surgical phase recognition

The classification results in this model were as follows: 90.7% for CCC, 94.5% for nuclear extraction, and 97.9% for others, with a mean response rate of 96.5%.

**Figure 4:**
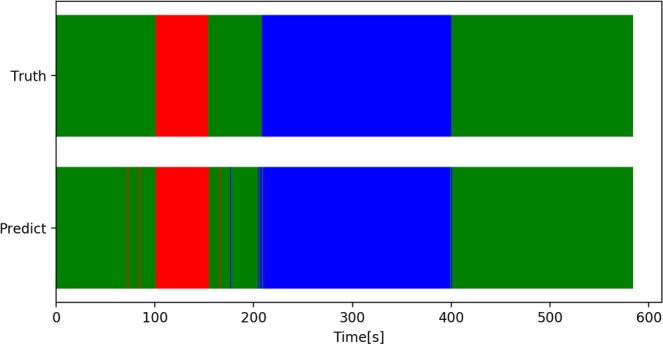
Examples of surgical phase recognition results. “Truth” represents the results identified by an ophthalmologist, and “Determination” represents the results recognized by the proposed method. The horizontal axis indicates the elapsed time on surgery. Colors red, blue, and green represent CCC, nuclear extraction, and others, respectively. Phase recognition errors occurred before and after the phase transition

The recognition error rates were as follows: misrecognized CCC as others, 9.3%; misrecognized nuclear extraction as others, 5.5%; and misrecognized others as CCC and nuclear extraction, 0.9% and 1.2%, respectively. The rate that the model could not distinguish between CCC and nuclear extraction was less than 0.01%. The results were shown in Table 5. Figure 4 shows an example of phase recognition for a video recording.

**Table 5:** Classification results of surgical phases of cataract surgery.

Truth/Classification	CCC [%]	Nuclear extraction [%]	Others [%]	Correct response rate [%]
CCC (n = 1725)	90.7	0	9.3	90.7
Nuclear extraction (n = 5995)	Less than 0.01	94.5	5.5	94.5
Others (n = 16095)	0.9	1.2	97.9	97.9
				Mean 96.5%

### Extraction of important surgical phases

**Figure 5:**
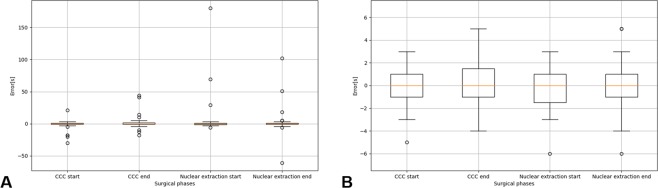
The view of the boxplot drawn based on errors of determination of start and end times of each surgical phase. (**A**) The entire view of the boxplot drawn based on errors of determination of start and end times of each surgical phase. (**B**) Enlarged view of the boxplot drawn based on errors of determination of start and end times of each surgical phase.

**Figure 6:**
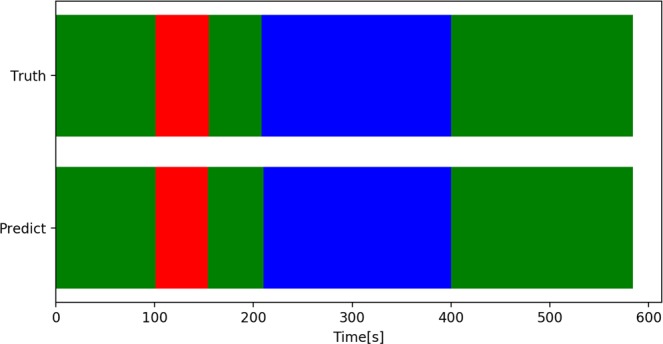
Examples of surgical phase extraction. (The video was the same as one used in Fig. 4). “Truth” represents the results identified by an ophthalmologist, and “Determination” represents the results recognized by the proposed method. The horizontal axis indicates the elapsed time on surgery. Colors red, blue, and green represent CCC, nuclear extraction, and others, respectively. The graphs indicate that the start and end times of all phases are accurate within a practical range.

The surgical phase recognition model described in Section 4.1 was used to determine the start and end times of each surgical phase. Performance evaluation was performed by finding the mean absolute error between the start and end times of each surgical phase recorded by the ophthalmologist and the start and end times determined by the proposed method. The results were as follows: CCC’s start and end times, 3.34 seconds and 4.43 seconds, respectively, and nuclear extraction’s start and end times, 7.21 seconds and 6.04 seconds, respectively, with a mean of 5.25 seconds as shown in Table 6. In addition, boxplots in Fig. 5(A,B) show that the start and end times of each surgical phase were determined with virtually no errors for most of the video recording. An example of surgical phases extracted from a video recording is shown in Fig. 6. The video was the same one used in Fig. 4.

**Table 6:** Determination of start and end times of each surgical phase.

Surgical phase	CCC start	CCC end	Nuclear extraction start	Nuclear extraction end	Mean
Average absolute error [sec.]	3.34	4.43	7.21	6.04	5.25
Standard deviation [sec.]	7.20	9.80	27.9	19.0	16.0

## Discussion and Conclusion

In the present study, real-time phase segmentation of cataract surgery videos was obtained. The convolution neural network was used to recognize important phases of cataract surgery in the surgical video at an average of 96.5%, and it determined the start and end times of these surgical phases with an error of about 5 seconds on average. Since the correct response data of the start and end times were obtained through visual observation of videos by several ophthalmologists instead of mechanical detection, deviation of several seconds from the true value is considered to be an acceptable range. Therefore, the model’s accuracy in phase segmentation was sufficiently high. Furthermore, the proposed method used 5 seconds of continuous video to recognize cataract surgical phases, which means that this method’s real-time phase recognition capability is greatly improved compared with previous studies^12,14,15^.

The successful phase segmentation of cataract surgery has great significance in forming the basis for more detailed automatic analysis within a phase. If the artificial intelligence engine first understands the “intent” of the current surgical maneuver and goes into further analysis, such as understanding the features of surgical instruments, such an analytical process is a natural algorithm, consistent with human reasoning, such as when a person chooses a tool for a certain purpose. The ability to analyze phases within 5 seconds also has significant implications for real-time performance. This is because the automatic analysis of surgical techniques during surgery would become the basis of abnormality detection, as in the case of self-driving cars. A number of surgical complications are caused by physicians who are at the development stage of the learning curve^1^. The present study was also regarded as groundwork in building a system that prevents complications by evaluating the surgical techniques of inexperienced surgeons during surgery.

In the present study, three cases have been classified, and the classifications were simpler compared to previous studies: 8-class classification^12^, 10-class classification^14^, and 14-class classification^15^. However, the CCC phase and nuclear extraction phase are both important surgical phases that cannot be regarded as identical to incision creation, cortical aspiration, or lens insertion. For example, from a surgical skills training perspective, the rate of surgical complications that developed in these two phases is clearly higher than in other phases^19^. The rate that the model confused the CCC phase with the nuclear extraction phase was almost zero, indicating a strong capability to distinguish phases. Because only surgical videos recorded at Tsukazaki Hospital were used, it was unclear how much influence it had on the results when shooting conditions and surgical instruments varied. However, 303 surgical videos included 17 surgeons, and there were 8 combination patterns of surgical techniques, surgical instruments, and lighting methods; therefore, the diversity of the dataset was secured to some extent. To our knowledge, no previous studies have detailed surgical patterns at the same level as the present study. For example, surgical methods or the number of surgeons were not described in the study of 8-class classification^12^. In the study of 10-class classification^14^, the following details were included: the number of surgeons, 10; the number of patients, 153– of which 33 patients had a bilateral surgery; the number of operating rooms, 2; and the operating rooms using a different camera. The study of 14-class classification^14^ was excellent in terms of performing recognition of 21 types of instruments; however, the surgical technique used was extracapsular cataract extraction (ECCE) using phacoemulsification and implantation of an intraocular lens (IOL), which is not a common phacoemulsification. In other words, the present study has competitive advantages in that it was conducted using a dataset containing the largest surgical patterns so far, and it succeeded in phase segmentation with high accuracy under a situation close to the clinical environment of general cataract phacoemulsification.

There are some cases including a large error. The large error of CCC start time occurred when viscoelastic substances were injected. The injector was misdiagnosed as cystotome. The large error of nuclear extraction start time occurred when the movies during nuclear extraction was very blurry. The large delays of determining CCC and nuclear extraction end time occurred when the surgeons do nothing after CCC. Increasing the amount of data and improving the training method would prevent such errors.

The present study was able to achieve a real-time phase segmentation of cataract surgery, using a practical clinical dataset including a wide variety of surgical techniques. Although there were only three phases, two of the most important cataract surgery techniques to train, CCC and nuclear extraction, were clearly segmented. The present study aimed to develop a risk prediction system for intraoperative complications of CCC and nuclear extraction based on the model. At the same time, the present study further aims to form the basic foundations of a system that can be used for broader cataract surgery training and safety management by developing a real-time phase segmentation model that includes cortical aspiration and IOL insertion.
